# Supplementary material for: Relationships between psychosocial factors during pregnancy and preterm birth in Puerto Rico
Source: PLoS One. 2020 Jan 29;15(1):e0227976. doi: 10.1371/journal.pone.0227976 (PMC6988967; doi:10.1371/journal.pone.0227976)

**S1 File Fig A. Flow diagram indicating participant selection into final analytic sample.**

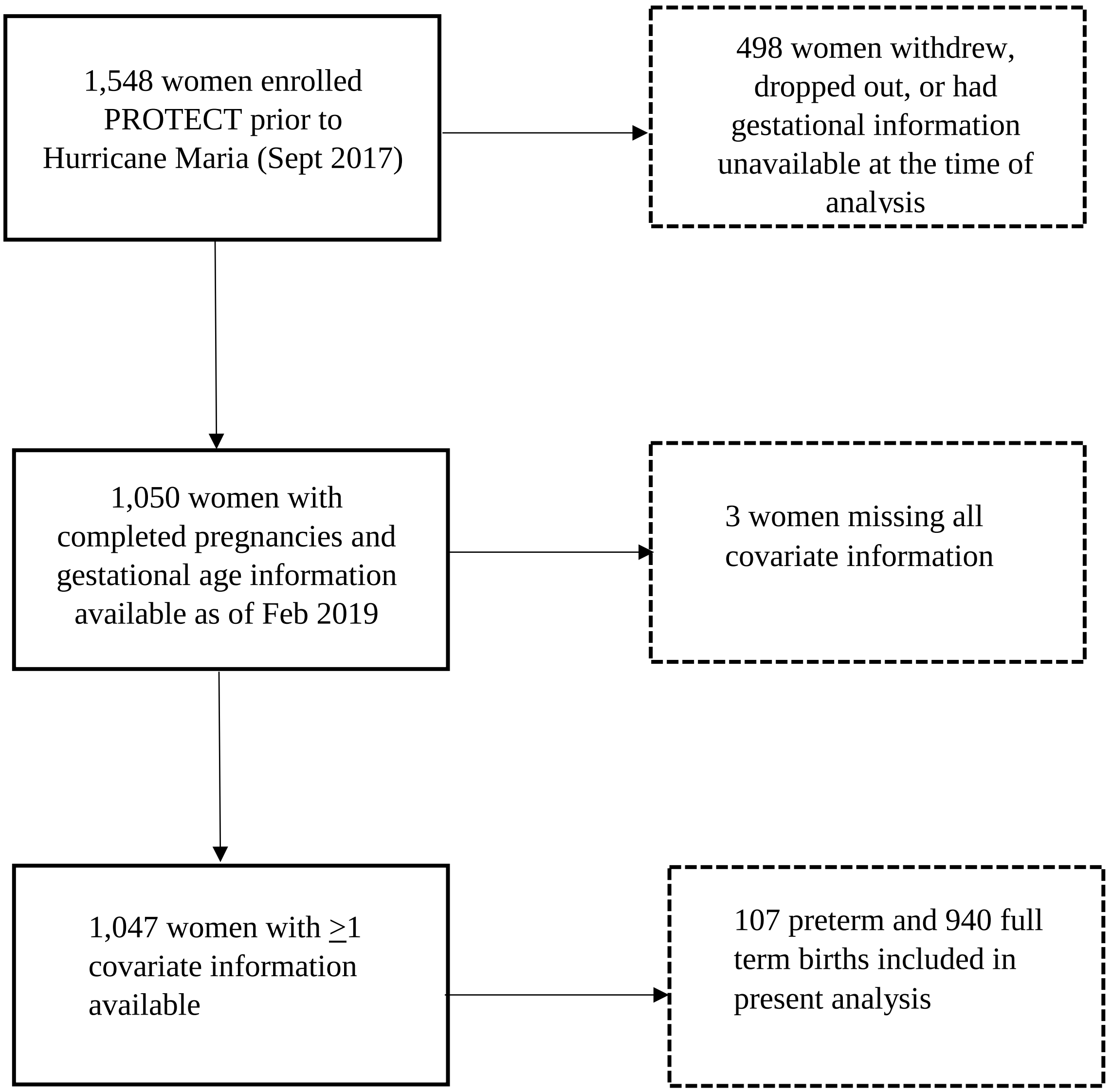

**S1 File Fig B. Distribution of Perceived Stress Scale (PSS) across demographic characteristics.**

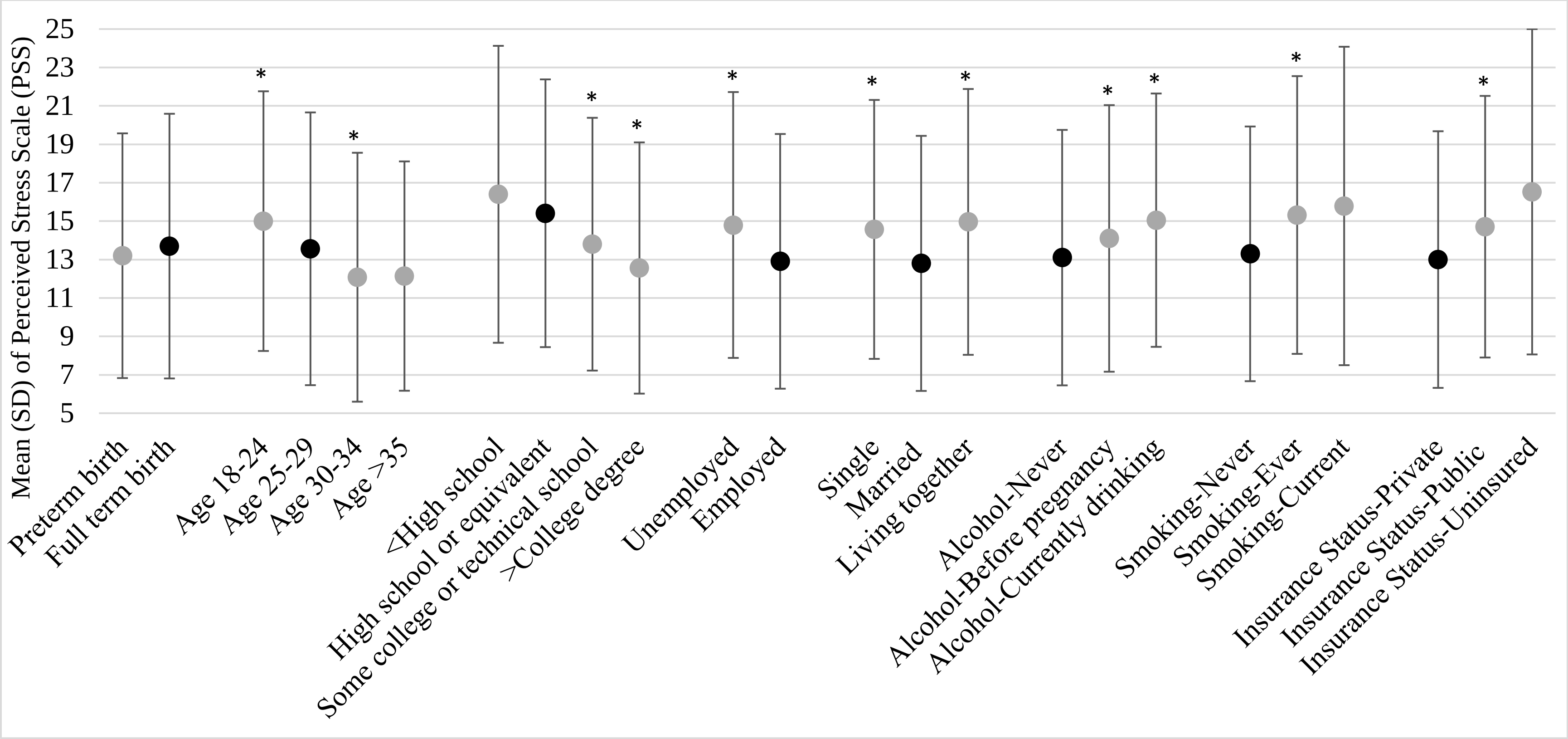


*Indicates p-value <0.05.

Abbreviations: SD, standard deviation.

Note: black indicates reference levels; p-values calculated from linear models.

**S1 File Fig C. Distribution of Life Experience Survey (LES) across demographic characteristics.**

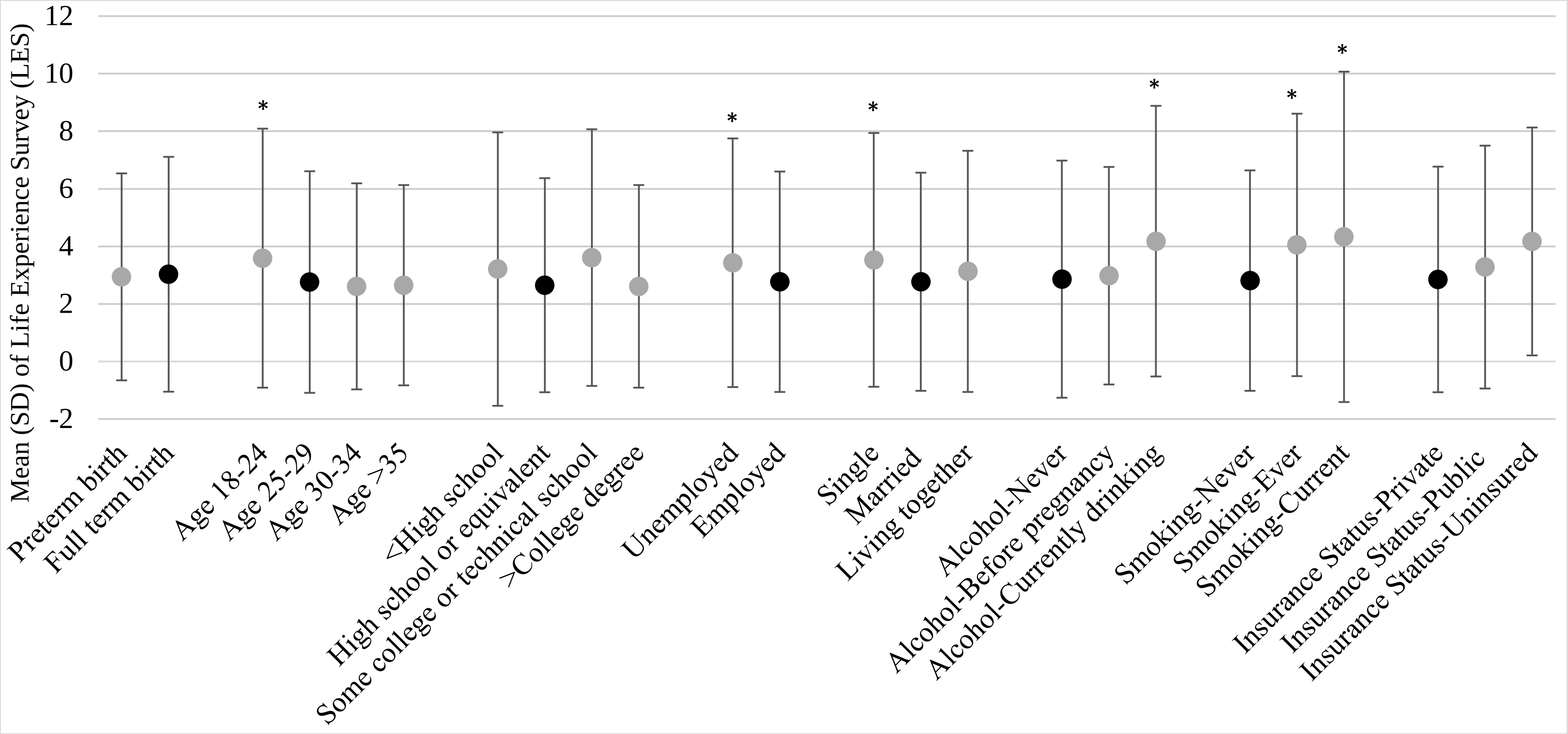


*Indicates p-value <0.05.

Abbreviations: SD, standard deviation.

Note: black indicates reference levels; p-values calculated from linear models.

**S1 File Fig D. Distribution of Center for Epidemiologic Studies-Depression (CES-D) across demographic characteristics.**

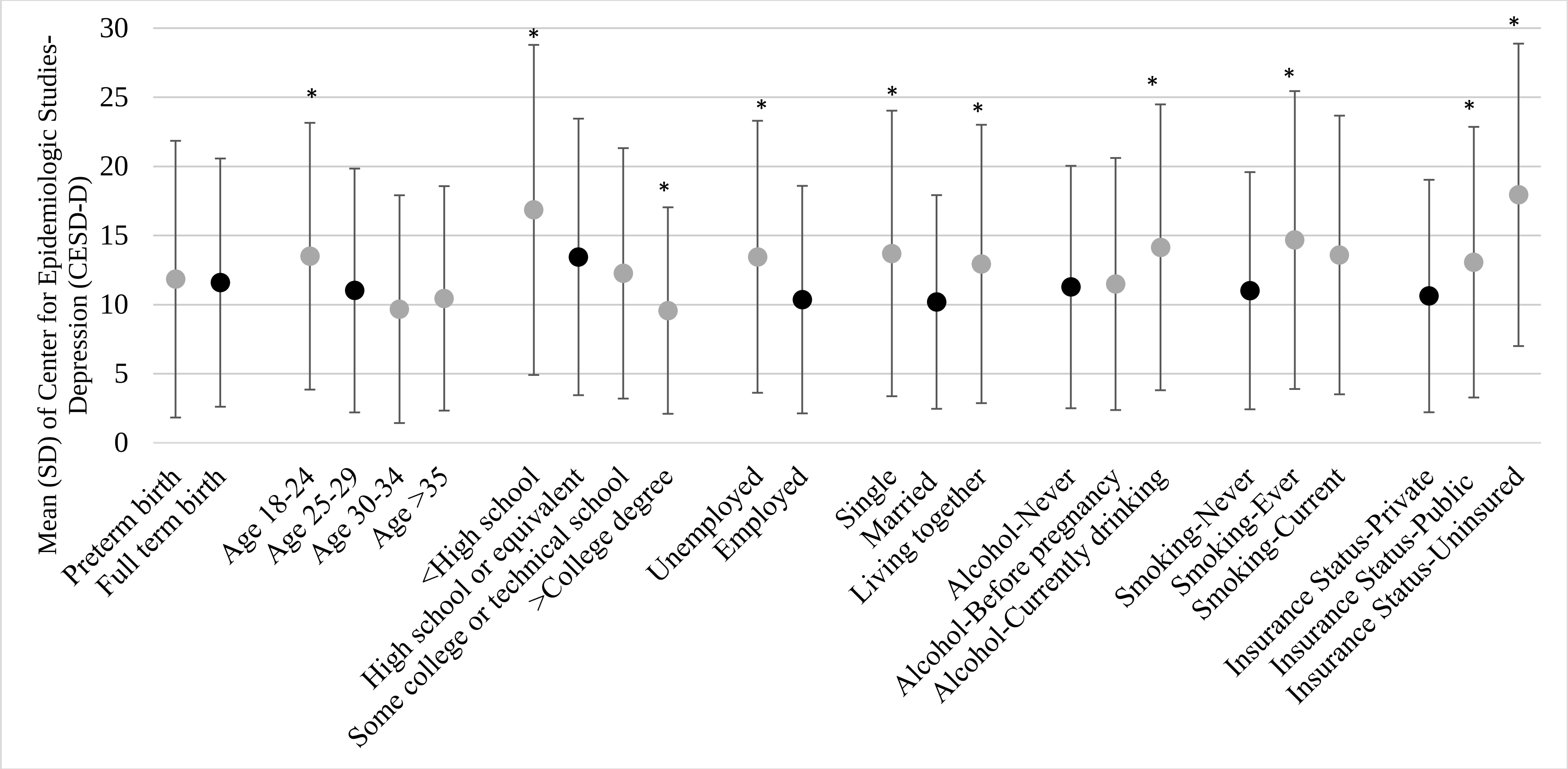


*Indicates p-value <0.05.

Abbreviations: SD, standard deviation.

Note: black indicates reference levels; p-values calculated from linear models.

**S1 File Fig E. Distribution of Neighborhood Perceptions (NP) across demographic characteristics**.

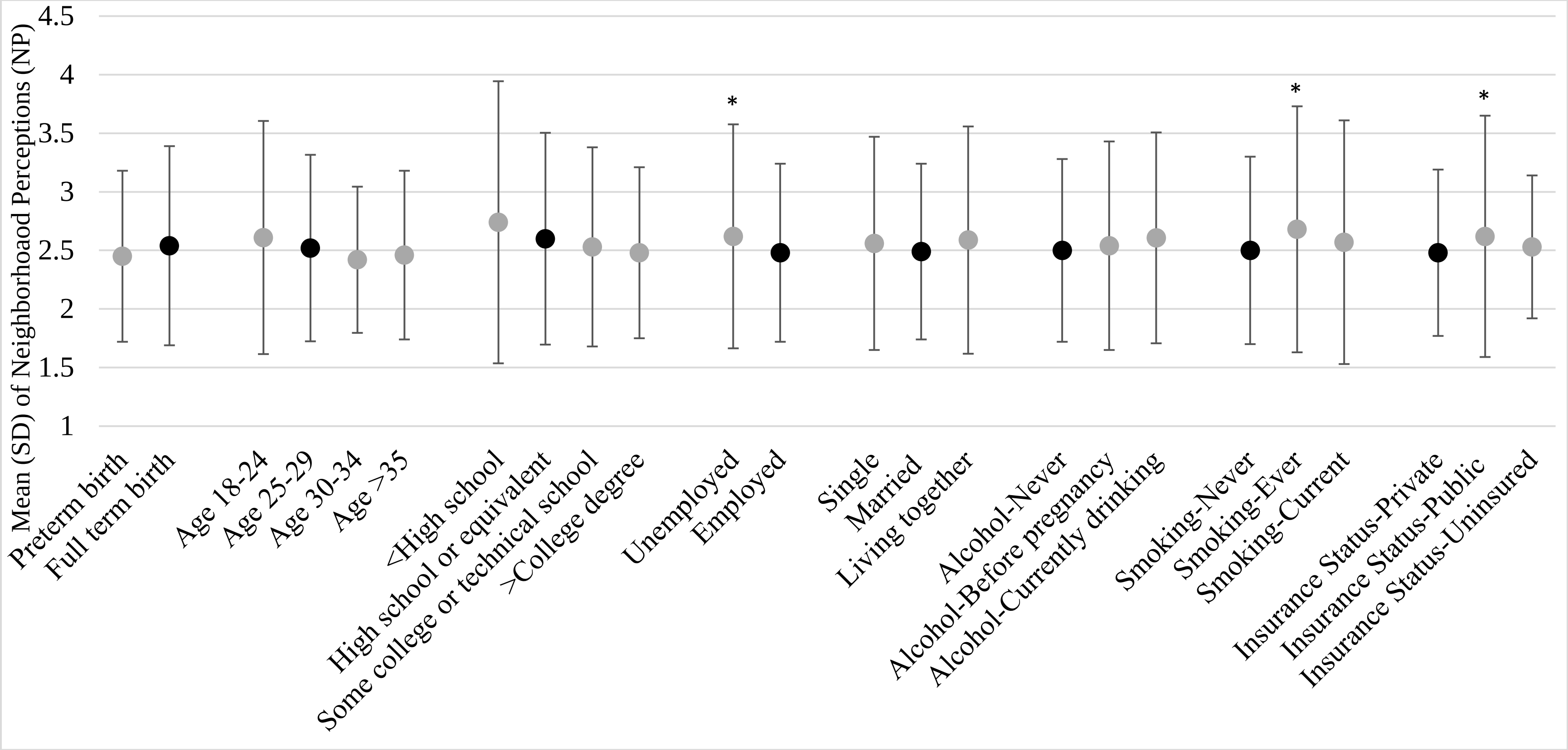


*Indicates p-value <0.05.

Abbreviations: SD, standard deviation.

Note: black indicates reference levels; p-values calculated from linear models.

**S1 File Fig F. Distribution of ENRICHD Social Support Instrument (ESSI) across demographic characteristics.**

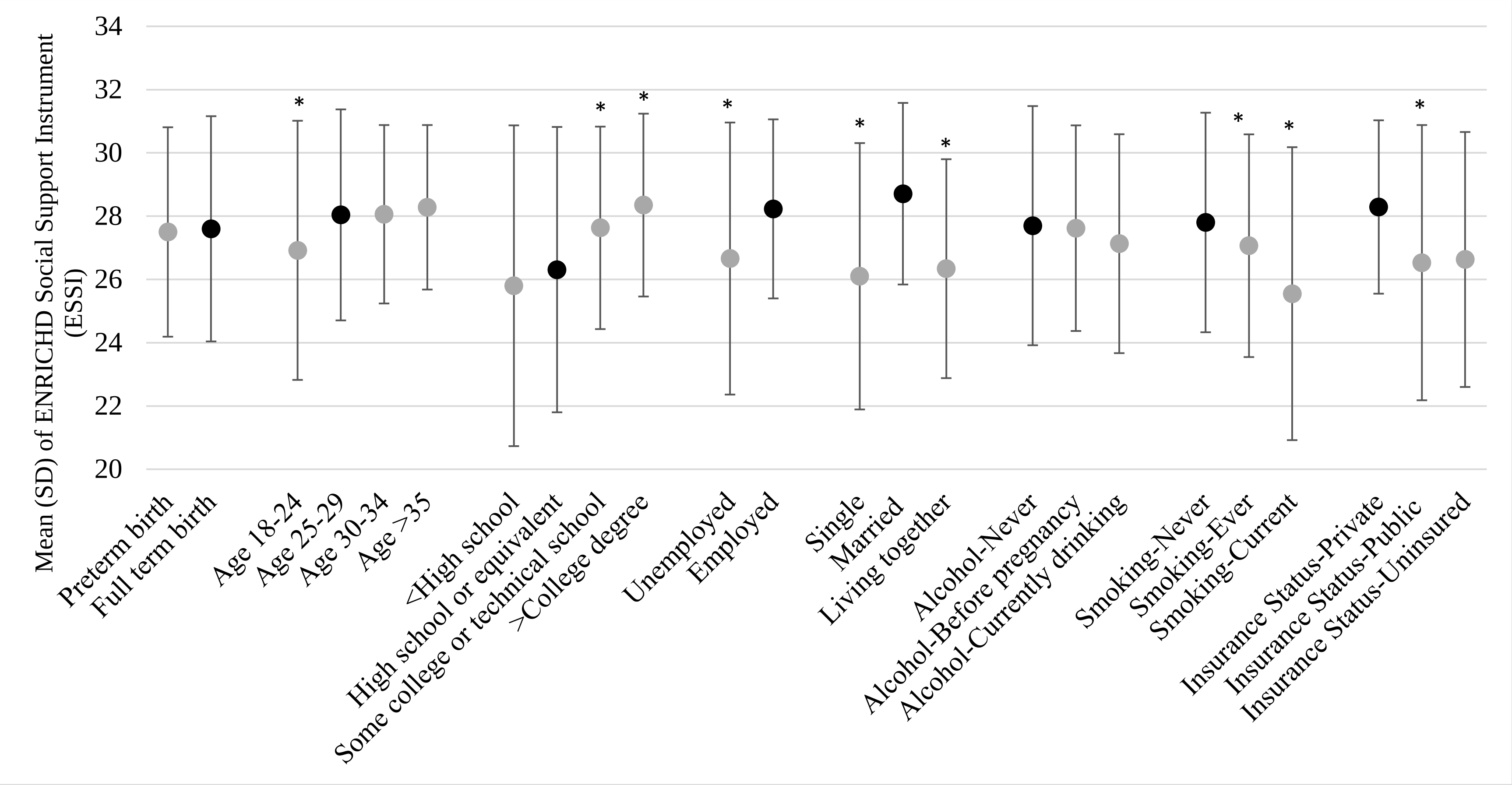


*Indicates p-value <0.05.

Abbreviations: SD, standard deviation.

Note: black indicates reference levels; p-values calculated from linear models.

**S1 File Fig G. Effect of perceived stress on depression moderated by social support.**

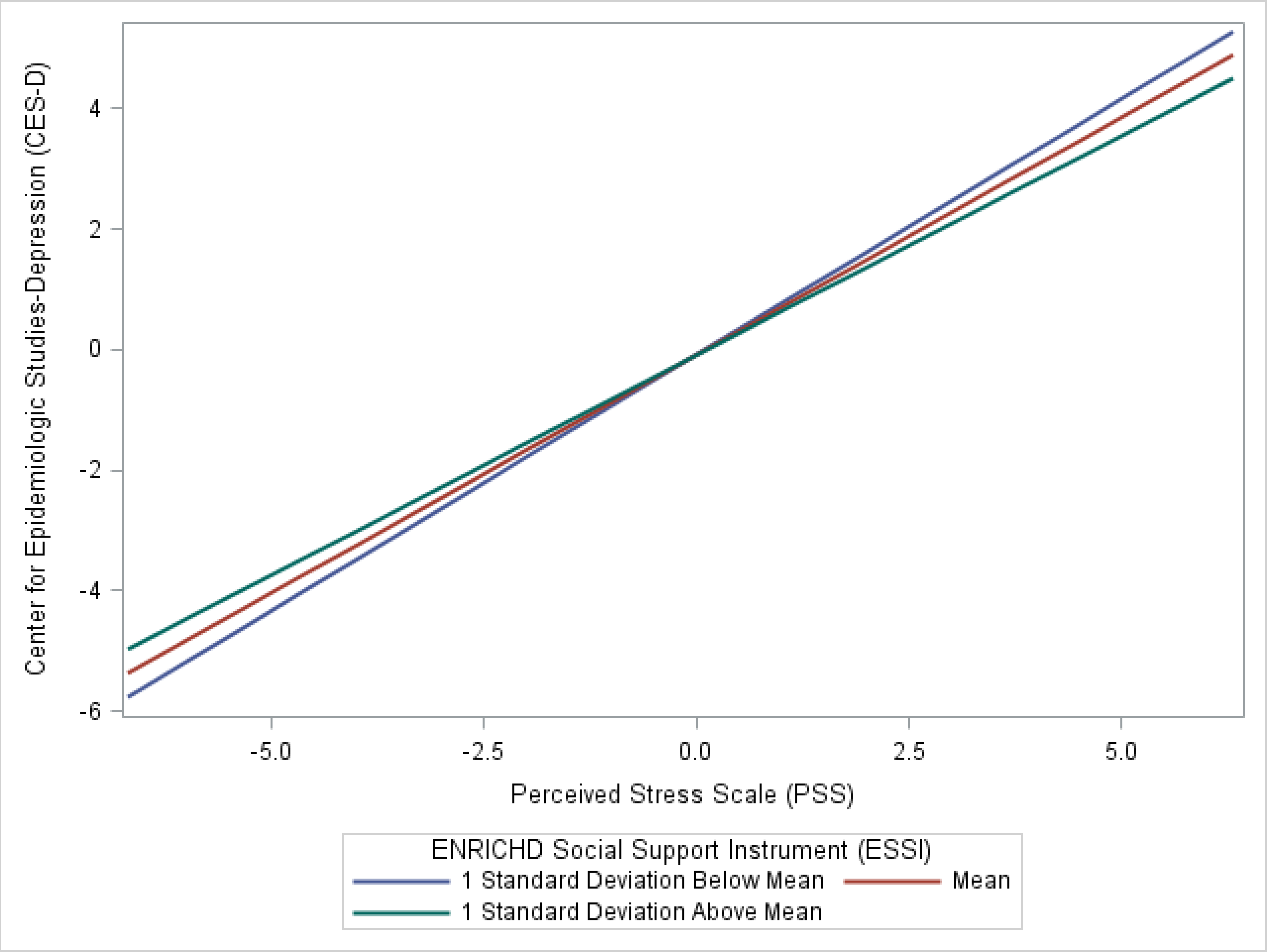

**S1 File Fig H. Full model including of all psychosocial stress measures, confounders, and effect modifiers.**

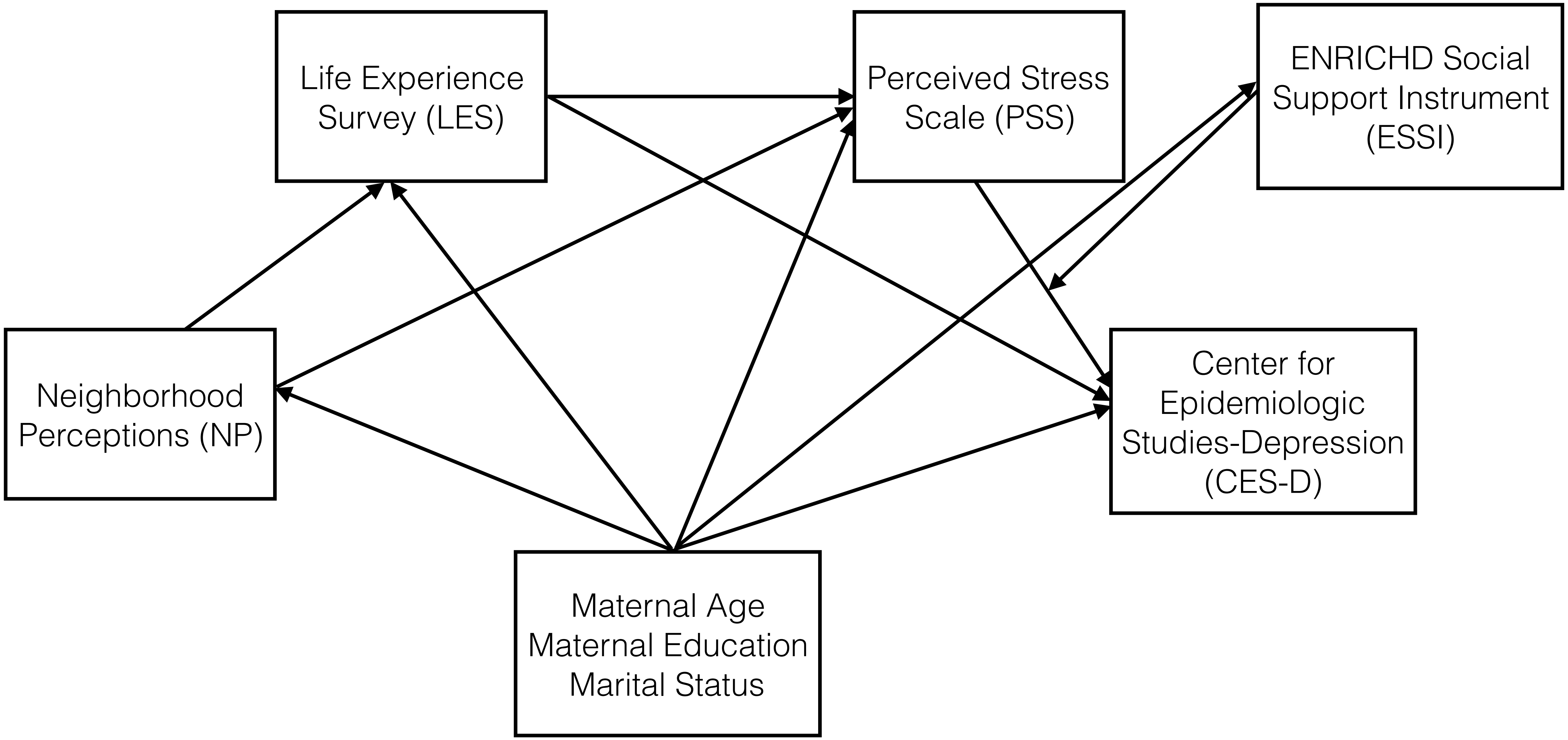

Supplement: S1 File — Fig A. Flow diagram indicating participant selection into final analytic sample. Fig B. Distribution of Perceived Stress Scale (PSS) across demographic characteristics. Fig C. Distribution of Life Experience Survey (LES) across demographic characteristics. Fig D. Distribution of Center for Epidemiologic Studies-Depression (CES-D) across demographic characteristics. Fig E. Distribution of Neighborhood Perceptions (NP) across demographic characteristics. Fig F. Distribution of ENRICHD Social Support Instrument (ESSI) across demographic characteristics. Fig G. Effect of perceived stress on depression moderated by social support. Fig H. Full model including of all psychosocial stress measures, depression, confounders, and effect modifiers. (PDF) [file pone.0227976.s001.pdf]
