# Supplementary material for: Relationships between psychosocial factors during pregnancy and preterm birth in Puerto Rico
Source: PLoS One. 2020 Jan 29;15(1):e0227976. doi: 10.1371/journal.pone.0227976 (PMC6988967; doi:10.1371/journal.pone.0227976)
Supplement: S2 File — Table A. Distribution of demographic characteristics among women with complete information on all psychosocial stress and depression measures (N = 841). Table B. Distribution of missingness between demographic characteristics and psychosocial stress and depression measures. (DOCX) [file pone.0227976.s002.docx]

**S2 File Table A. Distribution of demographic characteristics among women with complete information on all psychosocial stress measures (N=841).**

|  | N (%) |
| --- | --- |
| Preterm Birth |  |
| Yes | 79 (9.39) |
| No | 762 (90.6) |
| Maternal Age, years |  |
| 18-24 | 309 (36.8) |
| 25-29 | 268 (31.9) |
| 30-34 | 178 (21.2) |
| >35 | 85 (10.1) |
| Maternal Education |  |
| <High school | 56 (6.67) |
| High school or equivalent | 102 (12.2) |
| Some college or technical school | 302 (36.0) |
| >College degree | 379 (45.2) |
| Employment Status |  |
| Unemployed | 306 (36.6) |
| Employed | 529 (63.4) |
| Pre-pregnancy BMI |  |
| Underweight (<18.5 kg/m^2^) | 47 (5.88) |
| Normal (18.5-<25 kg/m^2^) | 403 (50.4) |
| Overweight (25-<30 kg/m^2^) | 210 (26.3) |
| Obese (>30 kg/m^2^) | 140 (17.5) |
| Marital Status |  |
| Single | 168 (20.0) |
| Married | 486 (57.8) |
| Living together | 187 (22.2) |
| Alcohol Use |  |
| Never | 422 (50.7) |
| Before pregnancy | 362 (43.5) |
| Currently drinking | 48 (5.77) |
| Smoking |  |
| Never | 710 (84.5) |
| Ever | 106 (12.6) |
| Current | 24 (2.86) |
| Insurance Status |  |
| Public | 542 (64.7) |
| Private | 282 (33.7) |
| Uninsured | 14 (1.67) |

Note: totals may not sum to 841 due to missing values.

Abbreviations: BMI, body mass index

**S2 File Table B. Distribution of missingness between demographic characteristics and psychosocial stress measures.**

|  | Missing ENRICHD Social Support Instrument (ESSI; N=86) | Missing Perceived Stress Scale (PSS; N=78) | Missing Center for Epidemiologic Studies-Depression (CES-D; N=109) | Missing Life Experience Survey (LES; N=71) | Missing Neighborhood Perceptions (NP; N=25) |
| --- | --- | --- | --- | --- | --- |
|  | N (%) | N (%) | N (%) | N (%) | N (%) |
| Maternal Age, years |  |  |  |  |  |
| 18-24 | 28 (32.6) | 27 (34.6) | 44 (40.4) | 35 (49.3) | 13 (52.0) |
| 25-29 | 22 (25.6) | 22 (28.2) | 25 (22.9) | 21 (29.6) | 6 (24.0) |
| 30-34 | 19 (22.1) | 19 (24.4) | 24 (22.0) | 8 (11.3) | 4 (16.0) |
| >35 | 17 (19.8) | 10 (12.8) | 16 (14.7) | 7 (9.86) | 2 (8.00) |
| Maternal Education |  |  |  |  |  |
| <High school | 6 (7.79) | 9 (11.8) | 11 (10.4) | 9 (10.0) | 1 (4.00) |
| High school or equivalent | 11 (14.3) | 10 (13.2) | 12 (11.3) | 13 (18.8) | 6 (24.0) |
| Some college or technical school | 26 (33.8) | 28 (36.8) | 42 (39.6) | 23 (33.3) | 10 (40.0) |
| >College degree | 34 (44.2) | 29 (38.2) | 41 (38.7) | 24 (34.8) | 8 (32.0) |
| Employment Status |  |  |  |  |  |
| Unemployed | 28 (36.4) | 31 (40.8) | 44 (41.1) | 31 (44.3) | 15 (60.0) |
| Employed | 49 (63.6) | 45 (59.2) | 63 (58.9) | 39 (55.7) | 10 (40.0) |
| Pre-pregnancy BMI |  |  |  |  |  |
| Underweight (<18.5 kg/m^2^) | 5 (6.58) | 7 (9.33) | 8 (7.62) | 8 (12.3) | 4 (17.4) |
| Normal (18.5-<25 kg/m^2^) | 36 (47.4) | 36 (48.0) | 49 (46.7) | 28 (43.1) | 9 (39.1) |
| Overweight (25-<30 kg/m^2^) | 23 (30.3) | 20 (26.7) | 28 (26.7) | 17 (26.2) | 4 (17.4) |
| Obese (>30 kg/m^2^) | 12 (15.8) | 12 (16.0) | 20 (19.0) | 12 (18.5) | 6 (26.1) |
| Marital Status |  |  |  |  |  |
| Single | 21 (27.6) | 25 (32.9) | 23 (21.5) | 14 (20.0) | 4 (16.0) |
| Married | 40 (52.6) | 37 (48.7) | 56 (52.3) | 35 (50.0) | 12 (48.0) |
| Living together | 15 (19.7) | 14 (18.4) | 28 (26.2) | 21 (30.0) | 9 (36.0) |
| Alcohol Use |  |  |  |  |  |
| Never | 38 (49.4) | 39 (51.3) | 52 (48.6) | 37 (53.6) | 17 (68.0) |
| Before pregnancy | 31 (40.3) | 30 (39.5) | 48 (44.9) | 27 (39.1) | 7 (28.0) |
| Currently drinking | 8 (10.4) | 7 (9.21) | 7 (6.54) | 5 (7.25) | 1 (4.00) |
| Smoking |  |  |  |  |  |
| Never | 64 (83.1) | 61 (80.3) | 86 (80.4) | 59 (84.3) | 20 (80.0) |
| Ever | 10 (13.0) | 11 (14.5) | 16 (15.0) | 9 (12.9) | 3 (12.0) |
| Current | 3 (3.90) | 4 (5.26) | 5 (4.67) | 2 (2.86) | 2 (8.00) |
| Insurance Status |  |  |  |  |  |
| Public | 31 (38.9) | 37 (51.4) | 52 (51.0) | 24 (51.1) | 0 (0.00) |
| Private | 46 (57.5) | 31 (43.1) | 47 (46.1) | 22 (46.8) | 1.00 (100.0) |
| Uninsured | 3 (3.75) | 4 (5.56) | 3 (2.94) | 1 (2.13) | 0 (0.00) |

Abbreviations: BMI, body mass index
